# Tea intake and total body bone mineral density of all ages: a Mendelian randomization analysis

**DOI:** 10.3389/fnut.2024.1289730

**Published:** 2024-02-21

**Authors:** Chen Xing, Yanrong Tan, Wentao Ni

**Affiliations:** ^1^Department of Gastroenterology, Institute of Digestive Diseases of PLA, The First Affiliated Hospital (Southwest Hospital), Army Medical University (Third Military Medical University), Chongqing, China; ^2^Beijing Key Laboratory of Genome and Precision Medicine Technologies, Department of Respiratory and Critical Care Medicine, Peking University People's Hospital, Beijing, China

**Keywords:** tea intake, total body bone mineral density (TB-BMD), causality, Mendelian randomization (MR) analysis, genome-wide association study (GWAS)

## Abstract

**Background:**

There is increasing evidence indicating that tea intake affects bone mineral density levels; however, the causality between tea intake and bone mineral density is inconclusive. This study aimed to assess the causal relationship between tea intake and total body bone mineral density (TB-BMD) through two-sample Mendelian randomization (MR) analysis.

**Methods:**

We conducted a two-sample MR approach to estimate the potential causal effects of tea intake on TB-BMD at all ages in a European population. The analyses were performed using summary statistics obtained for single-nucleotide polymorphisms (SNPs), identified from a genome-wide association meta-analysis of tea intake (*N* = up to 447,485 individuals) and from the GEnetic Factors for OSteoporosis (GEFOS) Consortium’s genome-wide association meta-analysis (*N* = up to 56,284 individuals), with baseline data collected in 2018 and populations derived from the European ancestry. The association between each SNP and TB-BMD was weighted by its association with tea intake, and estimates were combined mainly using an inverse-variance weighted meta-analysis. In addition, we explored the potential causal effects between green tea intake, herbal tea intake, and TB-BMD.

**Results:**

The MR analysis revealed that genetically determined tea intake exerts a causal impact on TB-BMD, with an odds ratio (OR) of 1.204 (95% CI: 1.062–1.366, *p* = 0.004), especially in the age group of 45–60 years (OR = 1.360, 95% CI: 1.088–1.700, *p* = 0.007). No horizontal pleiotropy and heterogeneity were observed. However, there was no causal effect of tea intake on TB-BMD in the age groups of 0–15, 15–30, 30–45, and over 60 years. In the subgroup analysis, when green tea intake was regarded as the exposure factor, no salient associations were found between green tea consumption and TB-BMD (IVW *p* = 0.368). Similarly, there was also no causal association between herbal tea intake and TB-BMD (IVW *p* = 0.264).

**Conclusion:**

The findings of this study support the evidence that tea consumption increases bone density and reduces the risk of osteoporosis in the age group of 45–60 years within the European population.

## Introduction

Bone mineral density (BMD) serves not only as an indicator of bone strength but also as a crucial measure for assessing osteoporosis. With the global population aging, the risk of osteoporosis is on the rise each year, particularly among the elderly and postmenopausal women. This has emerged as a significant public health concern, leading to an increased societal and economic burden (1). Osteoporosis exhibits a strong correlation with gender and age, influenced by factors such as race, height, body mass index, and unhealthy lifestyle choices (such as smoking, drinking, and coffee consumption) (2–6). It is noteworthy that the relationship between tea consumption and bone mineral density, and its potential role in osteoporosis, has been a topic often misunderstood. Previously, there was a misconception that drinking tea could lead to calcium loss and subsequently contribute to osteoporosis. This belief stems from the idea that caffeine in tea might hinder calcium absorption in the digestive tract and increase calcium excretion through urine (7–9). Additionally, the oxalates present in tea were thought to bind with calcium ions, resulting in a gradual loss of calcium from bones, thereby elevating the risk of fractures (10). Previous animal experiments and clinical studies showed a positive correlation between caffeine intake, particularly from coffee, and calcium loss, increasing the risk of osteoporosis and bone fractures (11–13). However, it is crucial to note that tea, unlike coffee, contains a more complex composition beyond just caffeine, and its impact on bone density may differ. Recently, an expanding body of observational research indicates that tea consumption does not contribute to calcium loss or a reduction in bone density (14, 15). Several studies have highlighted the potential benefits of tea in effectively enhancing bone density and preventing osteoporosis. However, it is challenging to establish conclusive evidence based solely on traditional observational studies. The causal relationship between tea intake and its impact on bone density remains unclear.

Mendelian randomization (MR) serves as an invaluable epidemiological tool, leveraging genetic variations linked to exposure factors to investigate the relationships between these genetic variants and outcomes, such as disease occurrence or mortality. Its core principle involves using genetic data as a means to effectively probe causal connections between a specific exposure and a particular outcome. Consequently, associations uncovered through MR are less prone to reverse causation and are less likely to be influenced by confounding factors. Moreover, MR, to a certain extent, addresses the limitations inherent in traditional randomized controlled trials (RCTs) and observational studies. In this study, a two-sample MR analysis was performed to explore the potential causal impact of tea intake on TB-BMD across all age groups, utilizing genetic data sourced from the GWAS database.

## Methods

### Study design and data source

**Figure 1:**
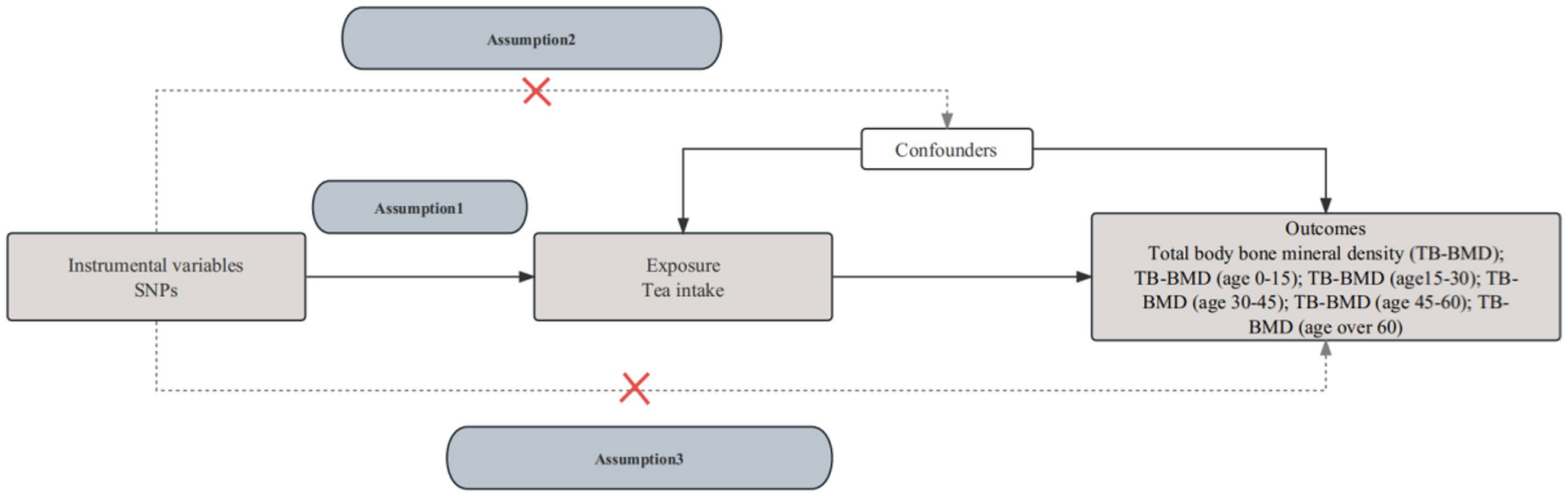
The flowchart of the Mendelian randomization assumptions.

In the MR analysis, it is crucial that the SNPs utilized as instrumental variables (IVs) satisfy three indispensable criteria. First, they must exhibit a strong association with the exposure variable (tea intake). Second, the selected SNPs should be independent of potential confounding factors. Finally, the instrumental variables should exert influence on the outcomes (total body bone mineral density, TB-BMD) solely through exposure without operating through alternative pathways (Figure 1).

We extracted reliable data sources based on the most comprehensive GWAS to explore the causal correlation between exposure and outcomes. Since all data were opened previously, and corresponding ethical review and informed consent had been obtained, we no longer needed any additional ethical approval.

Specifically, summary data pertaining to tea intake and TB-BMD across all age groups were sourced from the IEU OpenGWAS project.^1^ The data on tea intake was sourced from the United Kingdom Biobank (MRC-IEU), comprising a substantial sample size of 447,485 participants. Notably, the United Kingdom Biobank constitutes a cohort study encompassing individuals aged 40–69 years in the United Kingdom (16). Furthermore, the data on green tea and herbal tea intake were also extracted from the Medical Research Council Integrative Epidemiology Unit (MRC-IEU). The TB-BMD dataset, focusing on TB-BMD, was derived from a meta-analysis encompassing 30 genome-wide association studies (GWASs) and a total of 56,284 samples. These samples span a diverse age range, including 11,807 individuals aged 0–15 years, 4,180 individuals aged 15–30 years, 10,062 individuals aged 30–45 years, 18,805 individuals aged 45–60 years, and 22,504 individuals aged over 60 years (17) (Table 1). The TB-BMD serves as a reliable metric for evaluating osteoporosis and predicting fractures. To identify participants with osteopenia or osteoporosis, BMD *t*-scores were employed, adhering to the criteria set by the World Health Organization (18). Osteopenia was defined by a *t*-score ranging from −1 to −2.5, while osteoporosis was characterized by a *t*-score falling below −2.5 (18). Typically, dual-energy X-ray absorptiometry (DXA, Hologic Inc, Waltham, MA) was utilized to measure the total body bone density in grams per square centimeter (g/cm^2^). However, for pediatric individuals aged 0–15 years, the measurement method involves total body less head (TBLH) (17). In an effort to mitigate potential biases and minimize confounding factors, all subjects in this study belonged to the European ancestry group.

**Table 1:** Summary of GWAS in this study.

Exposure or outcomes	Consortia	Sample size	Number of SNPs	Ancestry	Year
Tea intake	MRC-IEU	447,485	9,851,867	European	2018
Green tea intake	MRC-IEU	64,949	9,851,867	European	2018
Herbal tea intake	MRC-IEU	64,949	9,851,867	European	2018
Total body bone mineral density	GEFOS	56,284	16,162,733	European	2018
Total body bone mineral density (age 0–15)	GEFOS	11,807	9,351,693	European	2018
Total body bone mineral density (age 15–30)	GEFOS	4,180	8,509,502	European	2018
Total body bone mineral density (age 30–45)	GEFOS	10,062	9,656,698	European	2018
Total body bone mineral density (age 45–60)	GEFOS	18,805	10,304,110	European	2018
Total body bone mineral density (age over 60)	GEFOS	22,504	11,932,096	European	2018

GWAS, Genome-wide association study; SNP, Single-nucleotide polymorphism; and GEFOS, Genetic factors for osteoporosis.

### Selection and validation of instrumental variables

An essential step was taken to meticulously select eligible IVs for tea intake based on the GWAS datasets. Specifically, the chosen single-nucleotide polymorphisms (SNPs) were required not only to exhibit a robust association with tea intake at a genome-wide significant level of *p* < 5 × 10^−8^ but also to undergo a clumping process, preventing biased results due to linkage disequilibrium (LD). The presence of SNPs in linkage equilibrium was detailed in the study conducted by Wu et al. (19). The criteria for the clumping procedure were *R*^2^ = 0.001 and window size = 10,000 kb. Moreover, we employed the PhenoScanner database^2^ to comprehensively scan for genetic variants linked to potential confounding factors. To address potential bias stemming from weak IVs, the F-statistic formula was employed for SNP selection, allowing the identification of SNPs with robust statistical power (20, 21). Subsequently, we extracted outcome data associated with the retained SNPs. Finally, to ensure the consistency of effect alleles between the exposure and outcome datasets, we harmonized the datasets by excluding palindromic and ambiguous SNPs with non-concordant alleles.

### Statistical analysis

The causal relationship between genetically predicted tea intake and TB-BMD was assessed through a two-sample MR analysis employing the Two-Sample MR package (22, 23). Various methods, such as MR-Egger, weighted median, inverse-variance weighted (IVW), simple mode, and weighted mode, were employed in the MR analysis to assess the relationship between tea consumption and TB-BMD across all ages. The IVW method, widely adopted in MR analysis, offers both fixed-effects and random-effects versions. As a meta-analytical technique, IVW combines the Wald estimates of causal effects for individual IVs, thereby providing comprehensive effect estimates of the exposure’s impact on the outcome (24). To enhance the robustness of the results, both IVW and MR-Egger assessments were utilized to evaluate the presence of heterogeneity. Heterogeneity was examined to discern variations among the IVs using Cochran’s *Q* statistic. A significance level of *p* < 0.05 indicated heterogeneity, prompting the application of the random-effects model for subsequent analyses; otherwise, the fixed-effects model was employed (25–27). Furthermore, the MR-Egger regression approach and the MR pleiotropy residual sum and outlier (MR-PRESSO) method were employed to identify and address pleiotropy effects (28). In the MR-Egger regression test, a significance level of *p*  < 0.05 indicated the presence of pleiotropy (29), while in the MR-PRESSO test, outliers were initially identified, followed by horizontal multiple-effects outlier correction. The subsequent assessment determined whether there was a significant difference in causal effects after removing the outliers (27). To further ensure the reliability of the analysis, a “leave-one-out” sensitivity analysis was conducted to explore the potential impact of individual SNPs on introducing bias and influencing the overall causal effect (19).

All analyses were carried out using two-sample MR (22) and MR-PRESSO (30) packages in software R (version 4.3.1).

## Results

After excluding SNPs with LD associated with tea intake based on parameters *r*^2^ and kb, conducting a search for surrogate SNPs, and retrieving SNPs linked to potential confounders through the PhenoScanner database, we identified 41 qualified SNPs selected as IVs for tea intake. Subsequently, we aligned the outcome data with the exposure SNPs, selecting corresponding SNPs associated with the exposure SNPs as IVs for further MR analyses (Supplementary Tables S1–S7). In our study, all IVs exhibited F-statistics exceeding 10, indicating minimal potential bias from weak IVs.

**Figure 2:**
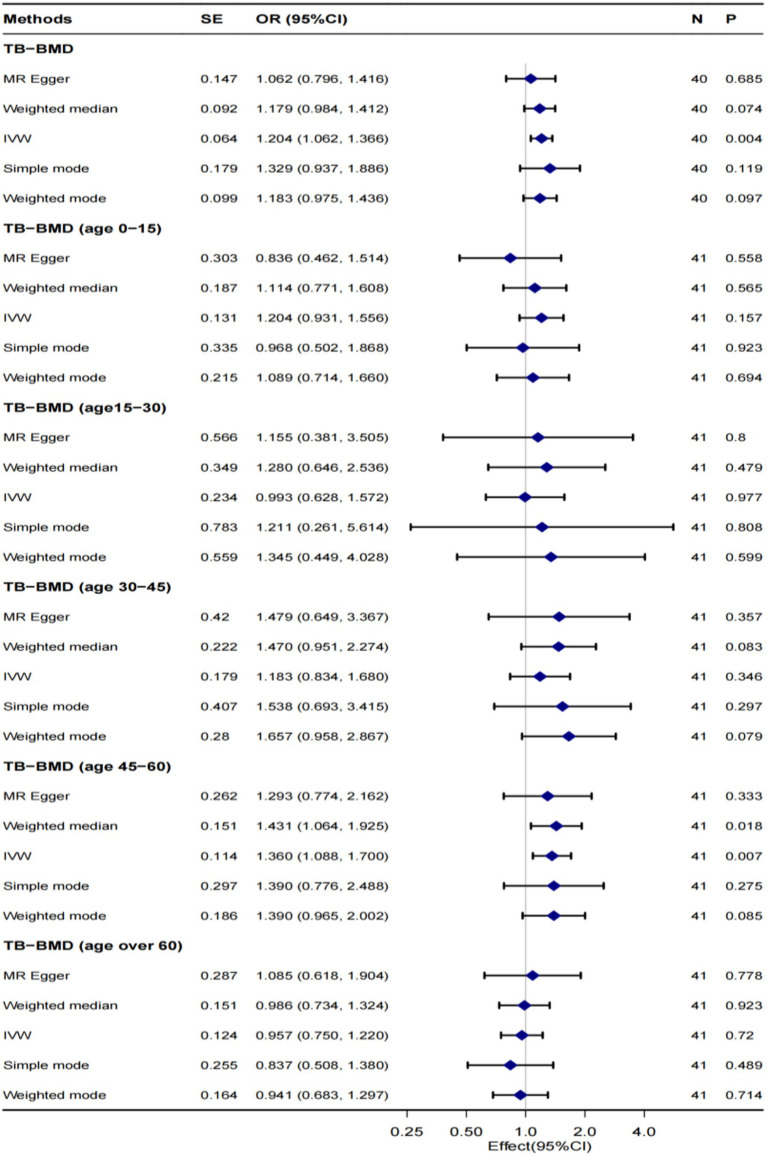
MR analysis of the causality of tea intake on TB-BMD at all ages. TB-BMD, Total body bone mineral density; IVW, Inverse-variance weighted; SNP, Single-nucleotide polymorphism; OR, Odds ratio; and CI, confidence interval.

Figure 2 illustrates the MR outcomes investigating the causal connection between genetically predicted tea intake and TB-BMD across all age groups (0–15, 15–30, 30–45, 45–60, and over 60 years) using different methods. The IVW method, as the primary analytical approach, revealed a robust causal relationship between genetically predicted tea intake and TB-BMD (OR 1.204, 95% CI 1.062–1.366, *p* = 0.004), especially in the age group of 45–60 years (OR 1.360, 95% CI 1.088–1.700, *p* = 0.007). However, no causal relationship was observed between tea intake and TB-BMD in the age groups of 0–15, 15–30, 30–45, and 60 years and above. The MR-Egger, weighted mode, simple mode, and weighted median approaches consistently demonstrated concordant outcomes.

**Figure 3:**
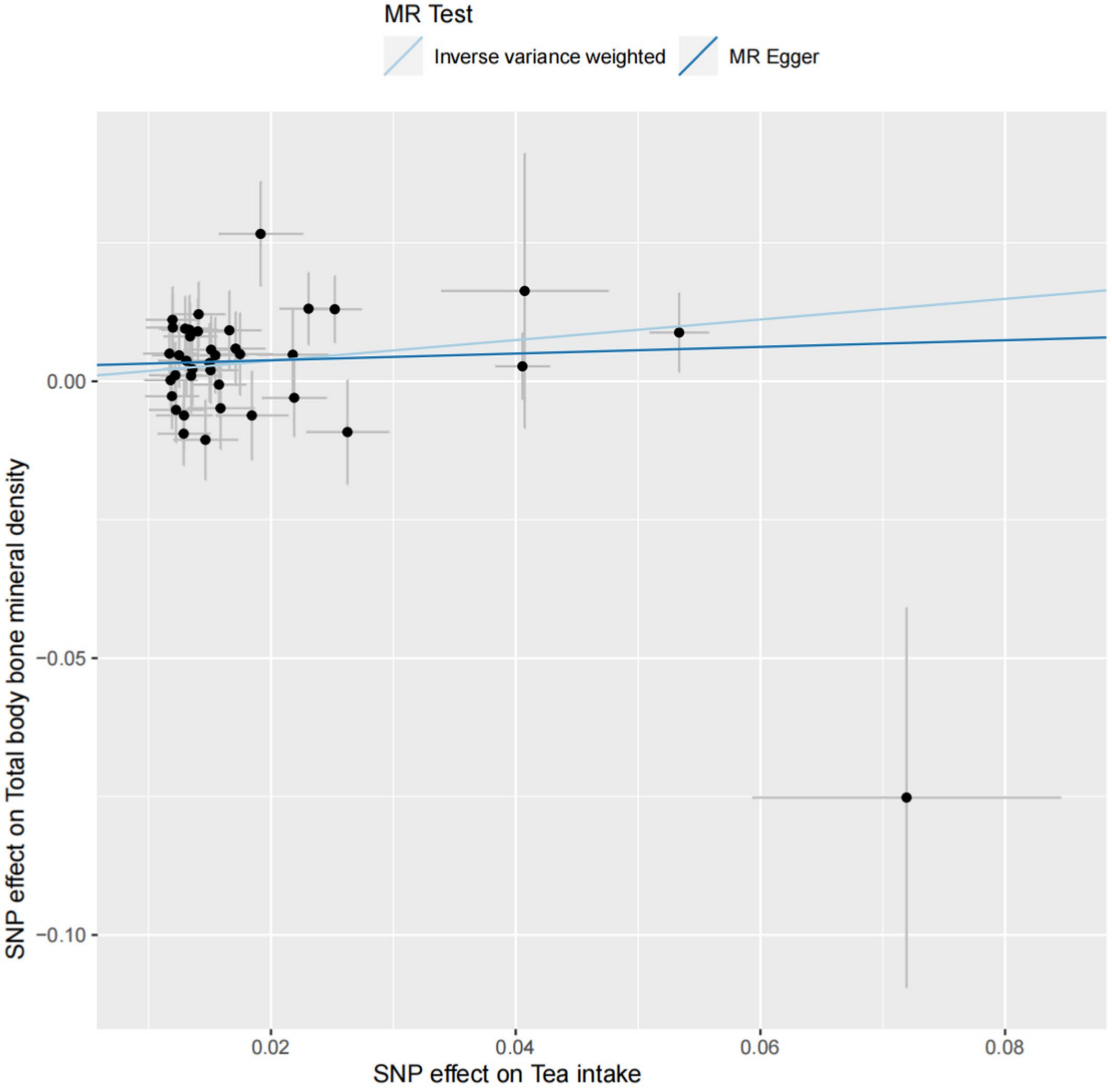
The scatter plot of the causal effect of tea intake on TB-BMD. Analyses were conducted using inverse-variance weighted and MR-Egger methods. The slope of each line corresponds to the causal estimates for two methods.

Significant heterogeneity was not observed between tea intake and TB-BMD (*p* > 0.05), and this lack of heterogeneity persisted within the age group of 45–60 years. The IVW and MR-Egger methods were employed to check for heterogeneity, and Table 2 presents Cochran’s *Q* and *p* values, respectively. The MR-Egger regression analysis did not reveal any evident directional pleiotropy (intercept = 0.003, *p* = 0.347; Figure 3), including among individuals aged 45–60 years (intercept = 0.001, *p* = 0.831) (Supplementary Figure S2). Moreover, the leave-one-out method analyzed single SNP risk, indicating that the association between tea intake and TB-BMD (including the age group 45–60) was not driven by any single SNP (Supplementary Figures S1, S2). The single SNP risk evaluation did not change significantly, reinforcing the robustness of the MR analysis. Additionally, the forest plot and the funnel plot are displayed in Supplementary Figures S1, S2. However, no causality was identified between tea consumption and TB-BMD across different age groups (0–15, 15–30, 30–45, and over 60 years) (Table 2; Supplementary Figures S3–S6).

**Table 2:** Heterogeneity test of the causal association between tea intake and TB-BMD in all ages.

Exposure	Outcome	MR-Egger		IVW	
		Cochran’s *Q*	*p*	Cochran’s *Q*	*p*
Tea intake	TB-BMD	47.532	0.115	48.696	0.115
Tea intake	TB-BMD (age 0–15)	37.799	0.433	39.610	0.398
Tea intake	TB-BMD (age 15–30)	41.145	0.335	41.238	0.373
Tea intake	TB-BMD (age 30–45)	54.608	0.031	55.119	0.036
Tea intake	TB-BMD (age 45–60)	44.237	0.193	44.292	0.223
Tea intake	TB-BMD (age over 60)	66.162	0.003	66.578	0.004

Furthermore, Tables 3, 4 present the MR results for the causal relationship between green tea consumption and TM-BMD, and herbal tea consumption and TB-BMD, respectively. The findings consistently indicate the absence of a causal relationship between green tea or herbal tea consumption and TB-BMD.

**Table 3:** MR analysis of the causality of green tea intake on TB-BMD.

Exposure	Outcome	Number of SNPs	Methods	OR (95%CI)	SE	*p*
Green tea intake	TB-BMD	21	MR Egger	1.000 (0.992,1.008)	0.004	0.955
			Weighted median	0.999(0.993,1.005)	0.003	0.725
			Inverse-variance weighted	0.998 (0.994,1.002)	0.002	0.368
			Simple mode	1.001 (0.991,1.011)	0.005	0.811
			Weighted mode	1.001 (0.992,1.010)	0.005	0.891

TB-BMD, Total body bone mineral density; SNP, Single-nucleotide polymorphism; OR, Odds ratio; and CI, Confidence interval.

**Table 4:** MR analysis of the causality of herbal tea intake on TB-BMD.

Exposure	Outcome	Number of SNPs	Methods	OR (95%CI)	SE	*p*
Herbal tea intake	TB-BMD	19	MR Egger	1.002 (0.980,1.025)	0.011	0.837
			Weighted median	0.998 (0.992,1.004)	0.003	0.496
			Inverse-variance weighted	0.998 (0.993,1.002)	0.002	0.264
			Simple mode	0.996 (0.985,1.007)	0.006	0.481
			Weighted mode	0.997 (0.987,1.007)	0.005	0.562

TB-BMD, Total body bone mineral density; SNP, Single-nucleotide polymorphism; OR, Odds ratio; and CI, Confidence interval.

## Discussion

We conducted comprehensive two-sample MR research to explore the causal effect of tea intake on BMD using GWAS summary data. All results showed that individuals with a genetic inclination toward consuming higher amounts of tea had a higher likelihood of experiencing increased BMD (OR = 1.204, *p* < 0.05). The findings remained consistent and robust even after conducting a thorough series of sensitivity analyses. Our research explored the causal relationship between tea consumption and bone density, proving that drinking tea does not affect calcium absorption or cause osteoporosis. On the contrary, it increases bone density and reduces the risk of osteoporosis, debunking the misconception that “drinking tea leads to osteoporosis.”

As predicted, our findings substantiated the results from several previous population-based observational studies and prospective studies, presenting that drinking tea contributes to the prevention of osteoporosis and an increase in BMD. Research on the relationship between tea consumption and bone density has yielded controversial findings. In 2017, Zhang et al. conducted a meta-analysis, revealing that tea consumption might enhance bone density and mitigate bone loss [odds ratio (OR): 0.66; 95% confidence interval (CI), 0.47–0.94; *p* = 0.02], particularly in areas such as the lumbar spine, hips, femoral neck, femoral trochanter, and femoral greater trochanter (all *p* values <0.05), indicating its potential in osteoporosis prevention (31). A 10-year tracking study in Sweden involving over 30,000 individuals suggested that consuming fewer than or up to four cups of tea daily exhibited no significant correlation with osteoporotic fractures among women (32). Another study assessed postmenopausal women in two Asian developing countries (Iran and India) for osteoporosis risk factors, indicating that consuming seven or more cups of tea per day was a significant protective factor against decreased bone density, ultimately reducing the risk of fractures (OR: 0.3; CI: 0.1–0.5) (33). Furthermore, research conducted by scholars at Texas Tech University in the United States proposed that long-term green tea consumption not only refrains from diminishing bone density but also may actually contribute to its increase (34).

These studies collectively point out that tea, due to its antioxidative, anti-inflammatory, bone-forming, and bone-resorption-inhibiting effects, can regulate bone metabolism, thereby preventing the onset of osteoporosis. This is particularly true for the main components of tea, the catechin compounds. After menopause, women experience a sharp decline in estrogen levels, which makes them susceptible to osteoporosis fractures. However, polyphenolic substances in tea can modulate skeletal health by exerting estrogen-like activities, enhancing osteoblast activity, and suppressing osteoclast-mediated bone resorption (35). According to Korean scholars, an investigation of data from 3,530 postmenopausal women between 2008 and 2011 indicated that women who consumed 1–3 cups of green tea daily exhibited significantly lower rates of osteopenia and osteoporosis compared to those who either did not consume green tea or consumed less than one cup daily (OR 1.81 and 1.85, 95% CI, 1.20–2.71; and 1.23–2.77) (36). Moreover, a 5-year prospective study involving 1,027 elderly women aged 7,085 years in Western Australia demonstrated that tea drinkers had a 2.8% higher total hip bone density compared to non-tea drinkers (*p* < 0.05) (37).

Based on the previous relevant reports overseas, some studies conducted in China have also confirmed the beneficial effects of tea consumption on increasing bone density and reducing the risk of fractures. A prospective study involving 453,625 adults found that tea drinkers had a significantly lower risk of fractures compared to non-tea drinkers (HR: 0.88; 95% CI: 0.83, 0.93) (38). Professor Huang further discovered that tea consumption was a significant independent predictor of bone density (*β* = 0.068, *p* < 0.05) (39), particularly among postmenopausal women. Long-term moderate tea drinking was found to be beneficial for skeletal health in postmenopausal women. However, the effect of tea drinking on bone health in men appears to be less significant, and the quantity of tea consumed has no apparent impact on skeletal health (40). In recent years, a study conducted by a team from Zhejiang University on postmenopausal women revealed significant differences in the impact of tea consumption on bone density between pre- and post-menopausal stages. Pre-menopausal tea drinkers showed significant increases in both total and regional bone density, with a more pronounced effect observed among those who consumed tea at least four times a week (41). Additionally, an epidemiological survey demonstrated a positive correlation between tea consumption and spine bone density, but with a non-linear increase as the duration of tea drinking increased (42).

There are several strengths in conducting the MR analysis. First, MR analysis effectively reduces the potential biases such as confounding factors and reverse causality. Second, utilizing SNPs associated with exposure enhances the precision of estimating potential causal relationships. Additionally, multiple evaluation methods and rigorous sensitivity analyses ensure the robustness of the results. Moreover, the direction of causal relationships in genetic correlations is deterministic. However, our study also has some limitations. First, the outcomes of MR may be influenced by different ethnicities, as heterogeneous populations can introduce biased effect estimates. This study is limited to individuals of European ancestry, and genetic variations among different ethnicities, countries, and regions may affect causal relationships. Second, MR studies cannot completely rule out hidden and unknown confounding factors, and future research should employ novel approaches and larger sample sizes to confirm the causal relationship between tea consumption and BMD. Finally, the MR analysis only provides statistical evidence for causal associations, and potential causal relationships should be comprehensively explored in conjunction with biological mechanisms.

## Conclusion

In conclusion, a genetic predisposition to tea intake was linked to increased BMD. As a result, moderate tea consumption in daily life does not warrant concern regarding calcium loss and osteoporosis. However, to validate the accuracy of these findings and to achieve a deeper understanding of the underlying pathophysiological mechanisms, further research employing advanced methods, larger GWAS datasets, and more comprehensive investigations is imperative.

## Data availability statement

The original contributions presented in the study are included in the article/Supplementary material, further inquiries can be directed to the corresponding author.

## Author contributions

CX: Data curation, Formal analysis, Investigation, Methodology, Supervision, Writing – original draft. YT: Investigation, Methodology, Writing – original draft. WN: Conceptualization, Writing – review & editing.

## Footnotes

^1^https://gwas.mrcieu.ac.uk/

^2^http://www.phenoscanner.medschl.cam.ac.uk/
