# Supplementary material for: Tea intake and total body bone mineral density of all ages: a Mendelian randomization analysis
Source: Front Nutr. 2024 Feb 21;11:1289730. doi: 10.3389/fnut.2024.1289730 (PMC10915007; doi:10.3389/fnut.2024.1289730)
Supplement: Supplementary file 1 [file Data_Sheet_1.docx]

**Supplementary Figure S1.**

**A B**

**
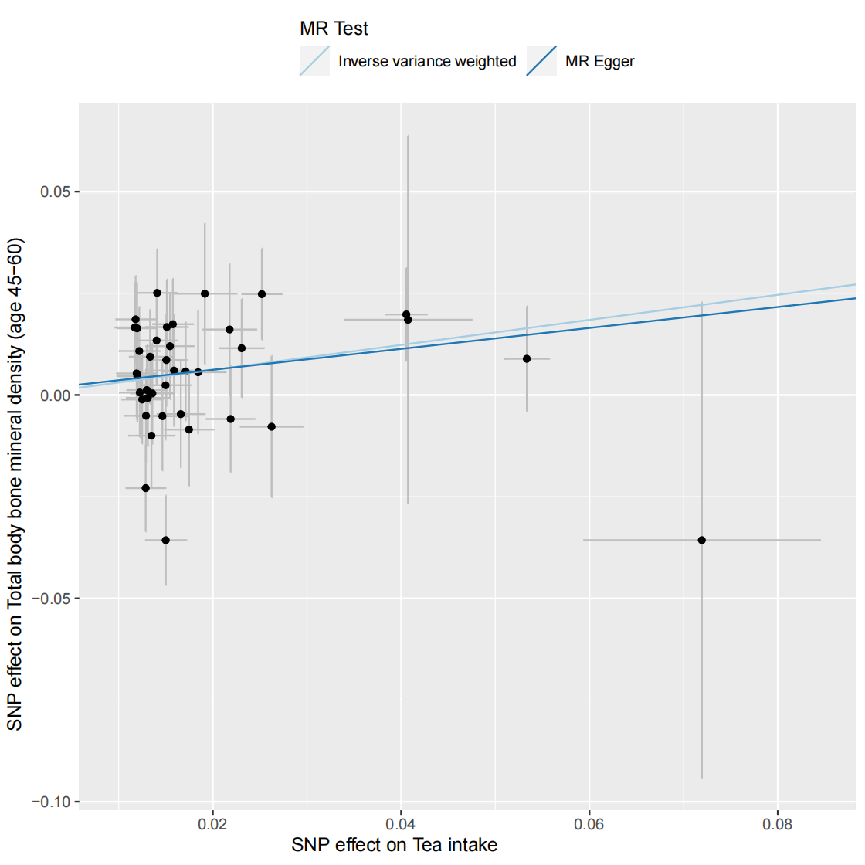

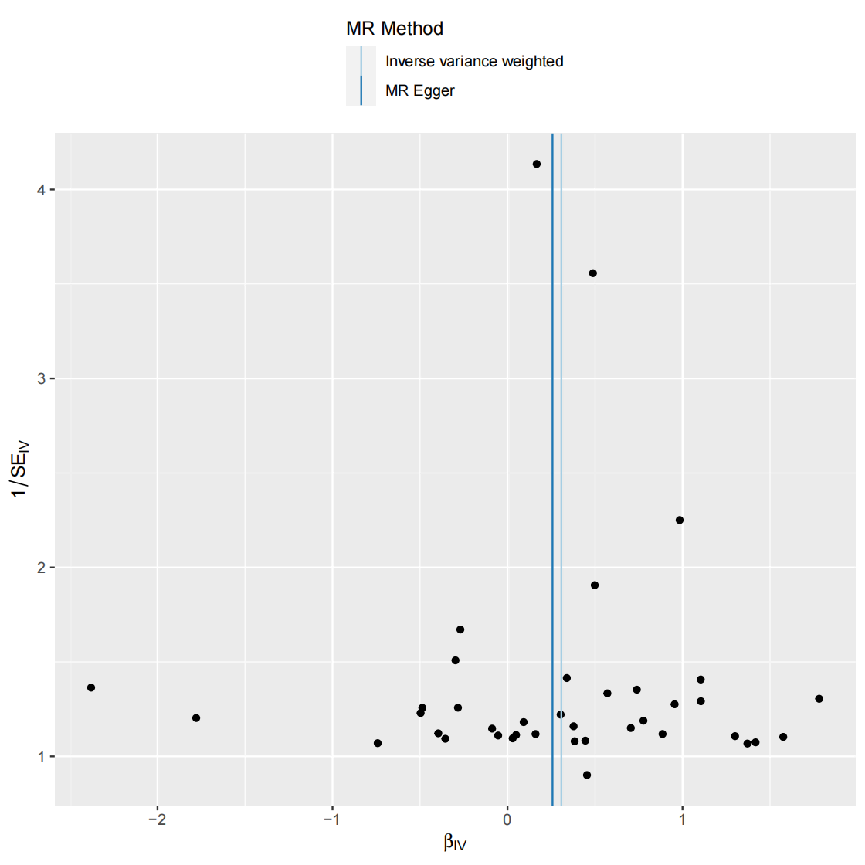
**

**C D**

**
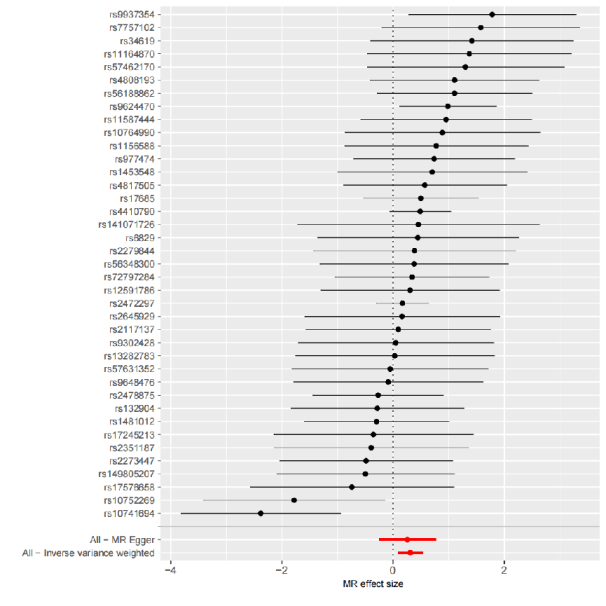

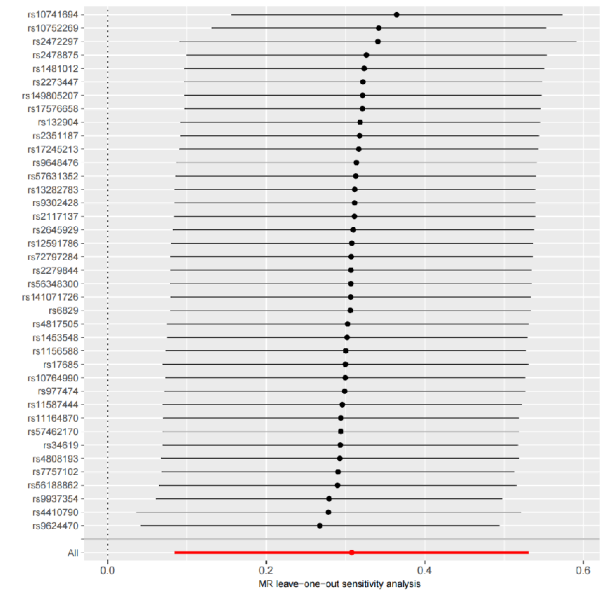
**

**(A)** The scatter plot of the causal effect of tea intake on TB-BMD (age 45-60). Analyses were conducted using the inverse variance weighted and MR-Egger. The slope of each line corresponding to the causal estimates for two methods. **(B)** The funnel plot for the impact of tea consumption on TB-BMD (age 45-60). The background of each SNP was outlined. **(C)** The leave-one-out sensitivity analysis plot for the impact of tea consumption on TB-BMD (age 45-60). The overall error lines remained stable after removing each SNP, reinforcing the reliability of the findings. All: the overall effect without removing SNPs. **(D)** The forest plot for the impact of tea consumption on TB-BMD (age 45-60). Individual SNP effects were computed independently, while the combined impact was assessed through MR-Egger and IVW techniques.

**Supplementary Figure S2.**

**A B**

**
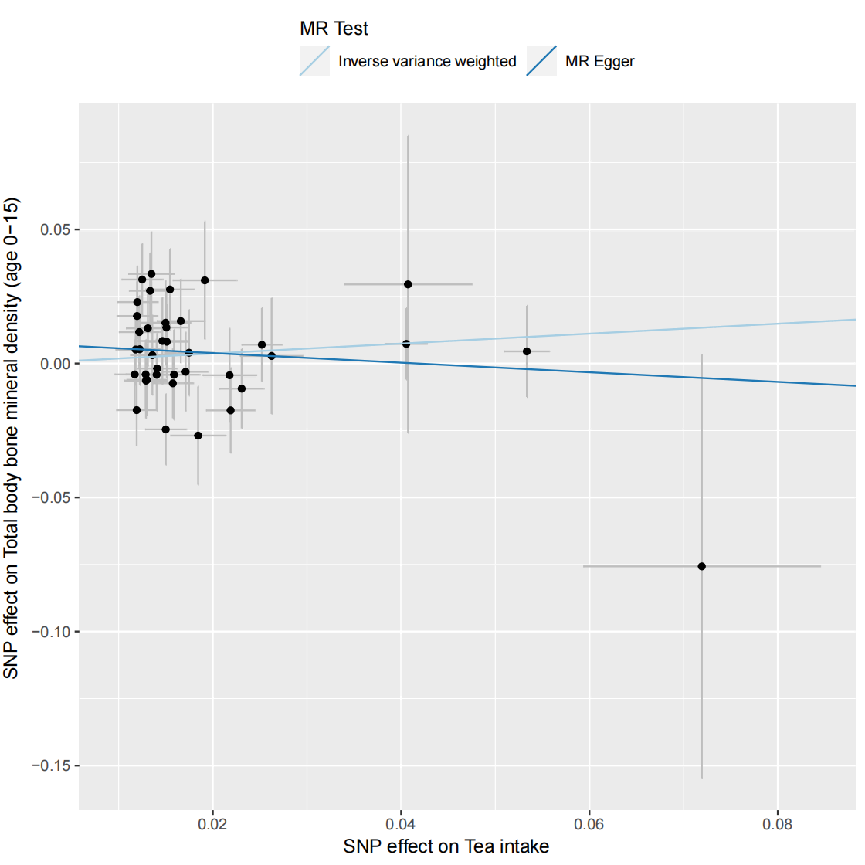

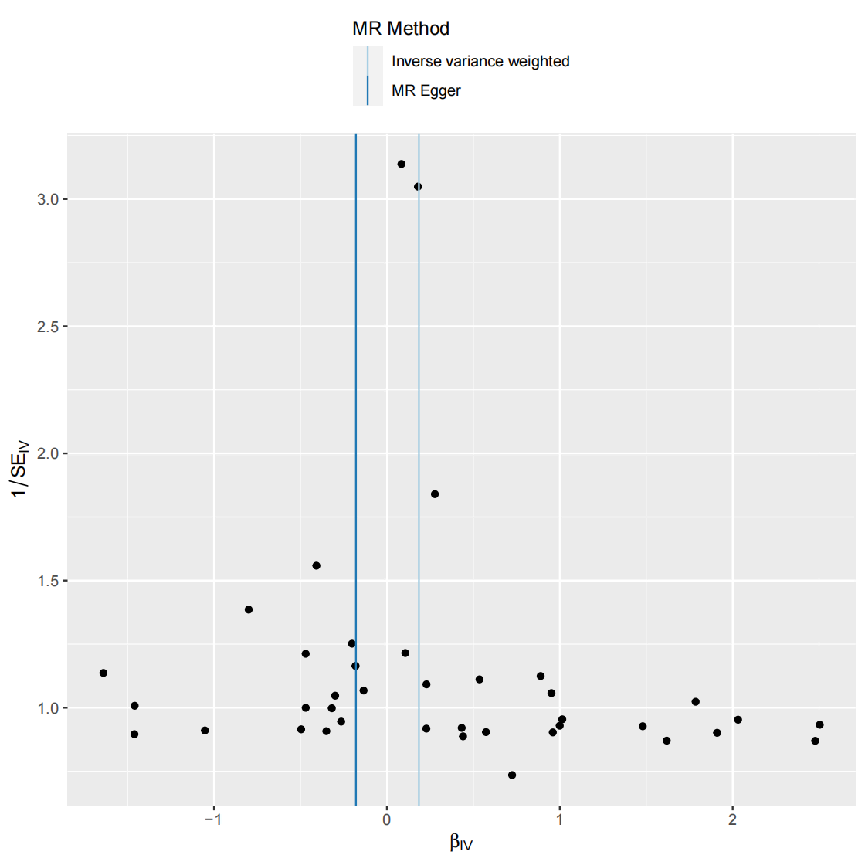
**

**C D**

**
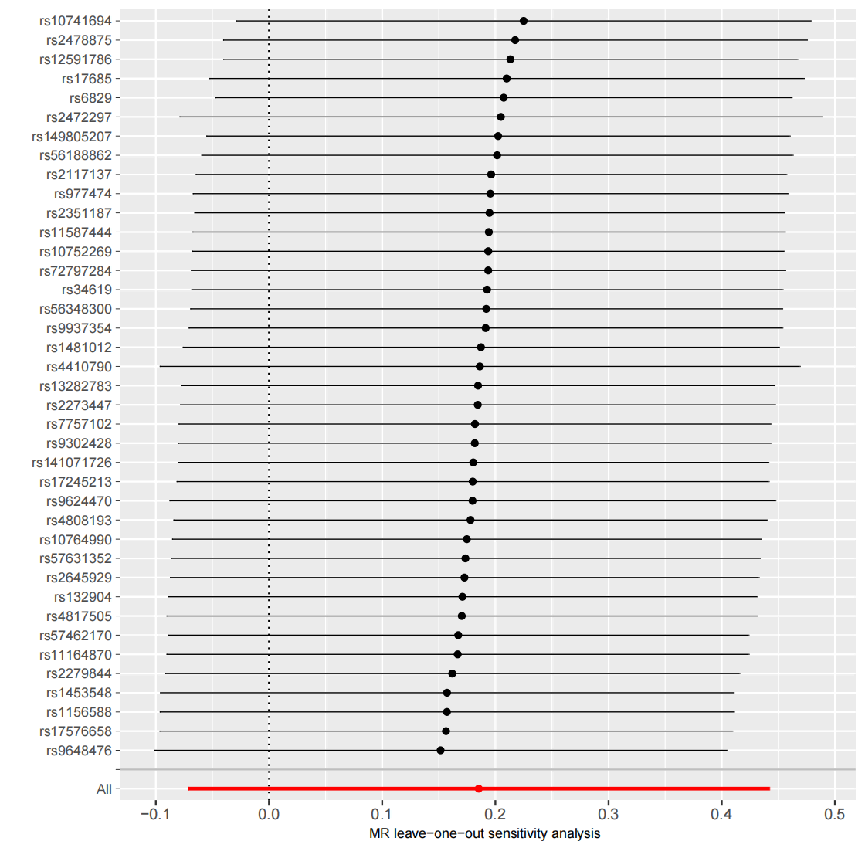

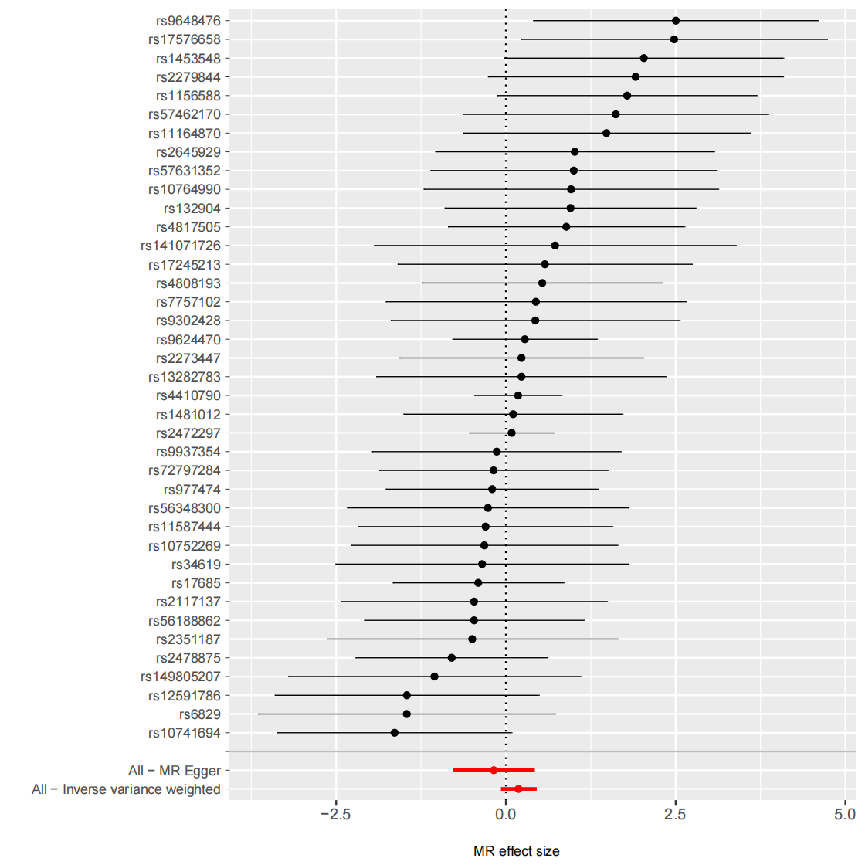
**

**(A)** The scatter plot of the causal effect of tea intake on TB-BMD (age 0-15). Analyses were conducted using the inverse variance weighted and MR-Egger. The slope of each line corresponding to the causal estimates for two methods. **(B)** The funnel plot for the impact of tea consumption on TB-BMD (age 0-15). The background of each SNP was outlined. **(C)** The leave-one-out sensitivity analysis plot for the impact of tea consumption on TB-BMD (age 0-15). The overall error lines remained stable after removing each SNP, reinforcing the reliability of the findings. All: the overall effect without removing SNPs. **(D)** The forest plot for the impact of tea consumption on TB-BMD (age 0-15). Individual SNP effects were computed independently, while the combined impact was assessed through MR-Egger and IVW techniques.

**Supplementary Figure S3.**

**A B**

**
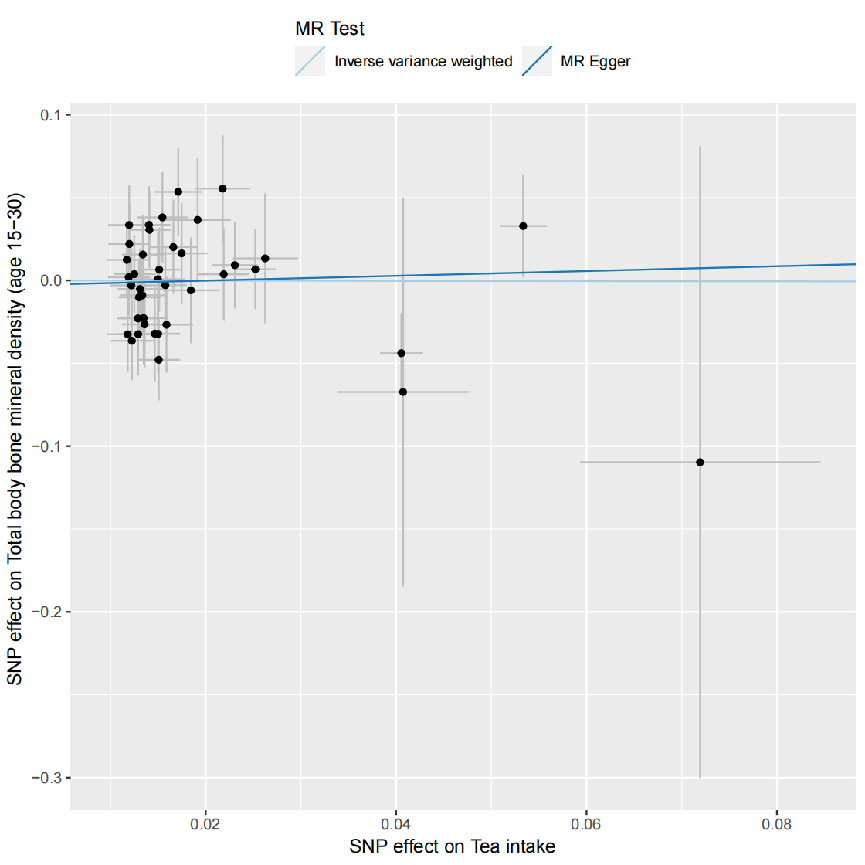

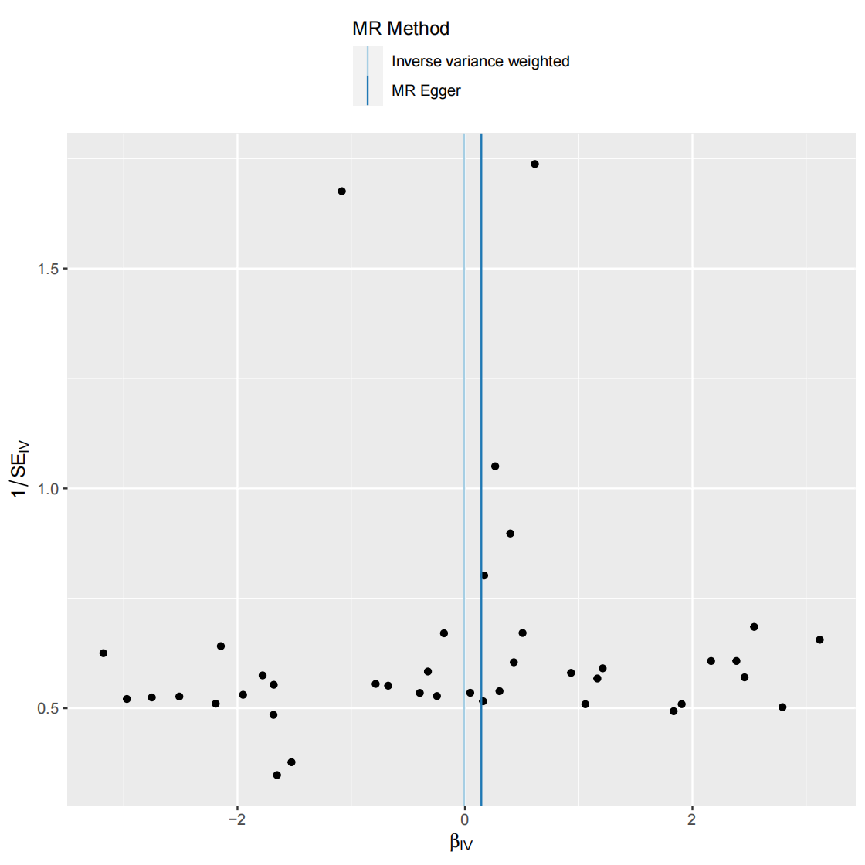
**

**C D**

**
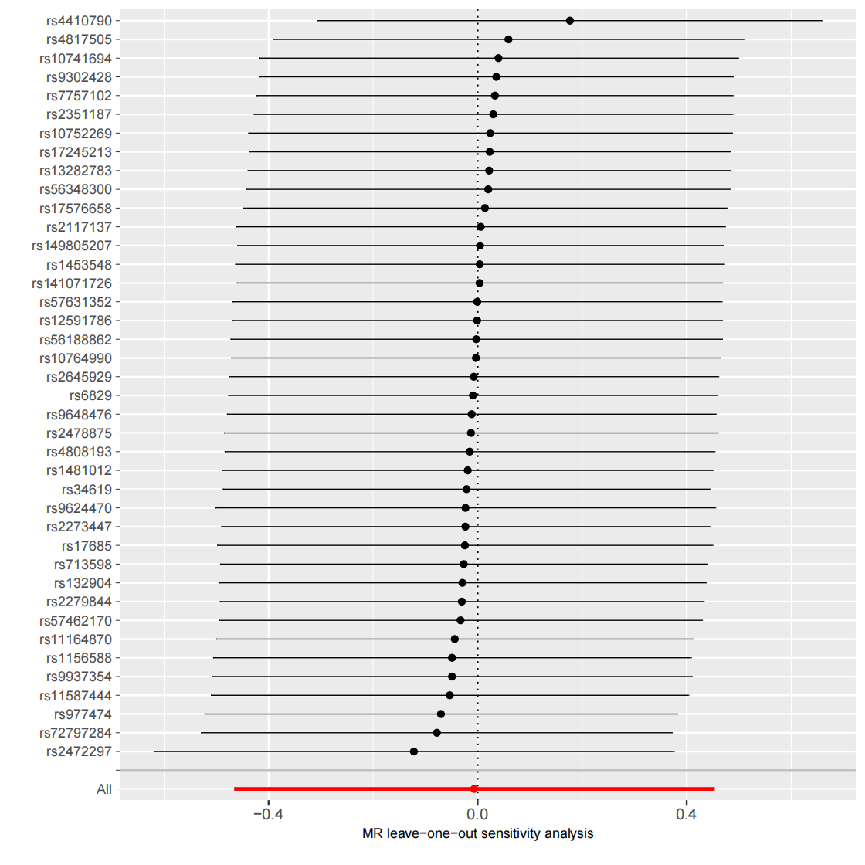

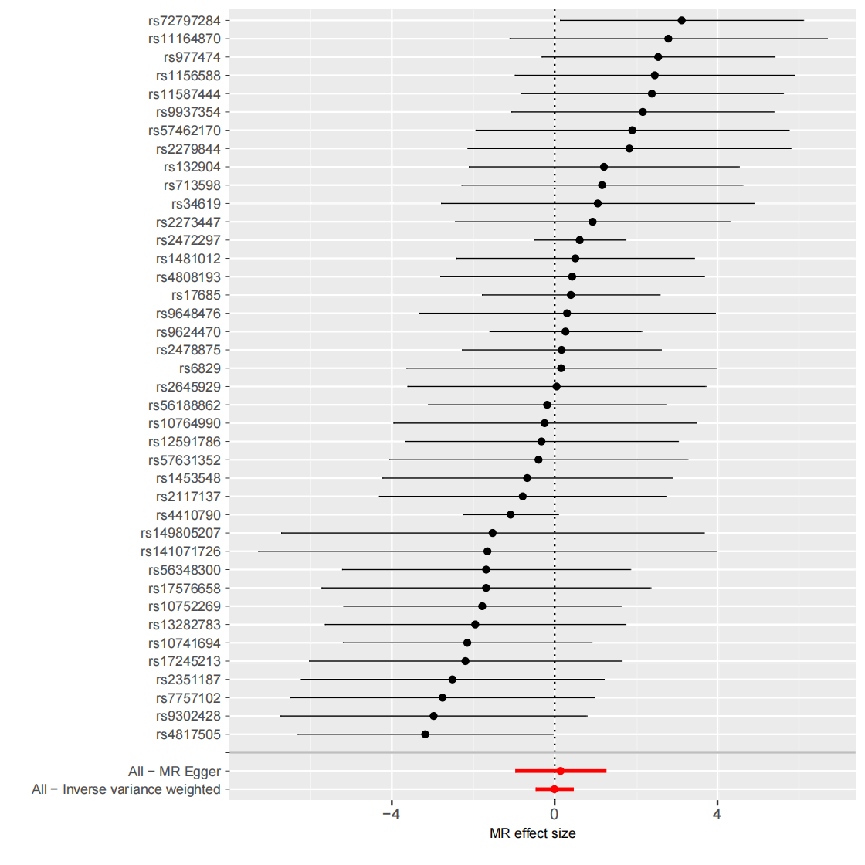
**

**(A)** The scatter plot of the causal effect of tea intake on TB-BMD (age 15-30). Analyses were conducted using the inverse variance weighted and MR-Egger. The slope of each line corresponding to the causal estimates for two methods. **(B)** The funnel plot for the impact of tea consumption on TB-BMD (age 15-30). The background of each SNP was outlined. **(C)** The leave-one-out sensitivity analysis plot for the impact of tea consumption on TB-BMD (age 15-30). The overall error lines remained stable after removing each SNP, reinforcing the reliability of the findings. All: the overall effect without removing SNPs. **(D)** The forest plot for the impact of tea consumption on TB-BMD (age 15-30). Individual SNP effects were computed independently, while the combined impact was assessed through MR-Egger and IVW techniques.

**Supplementary Figure S4.**

**A B**

**
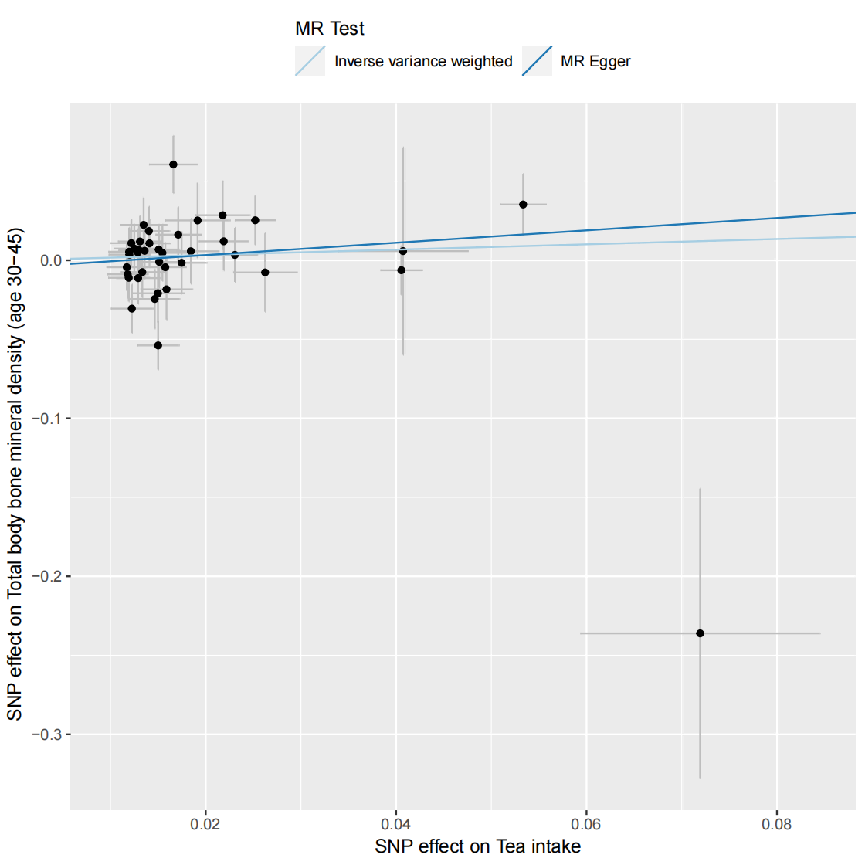

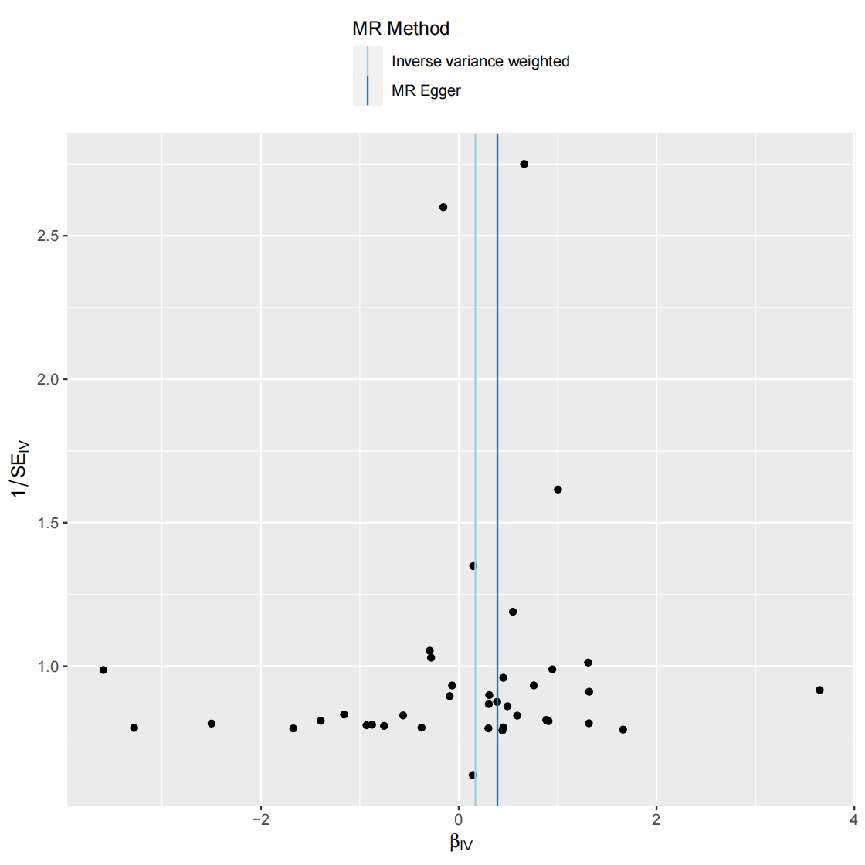
**

**C D**

**
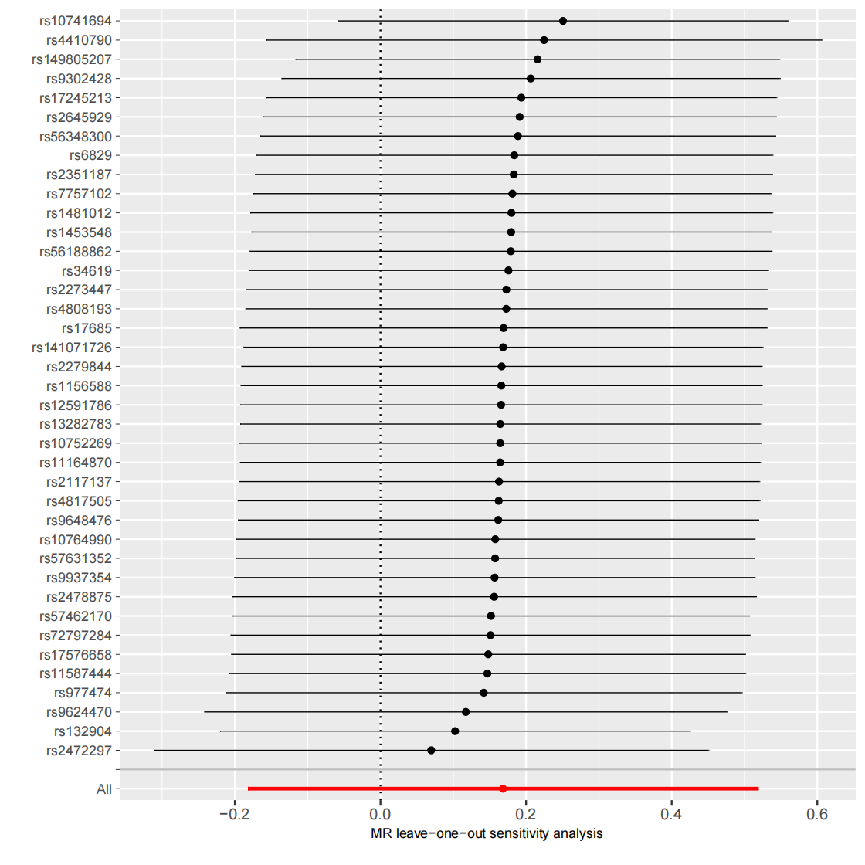

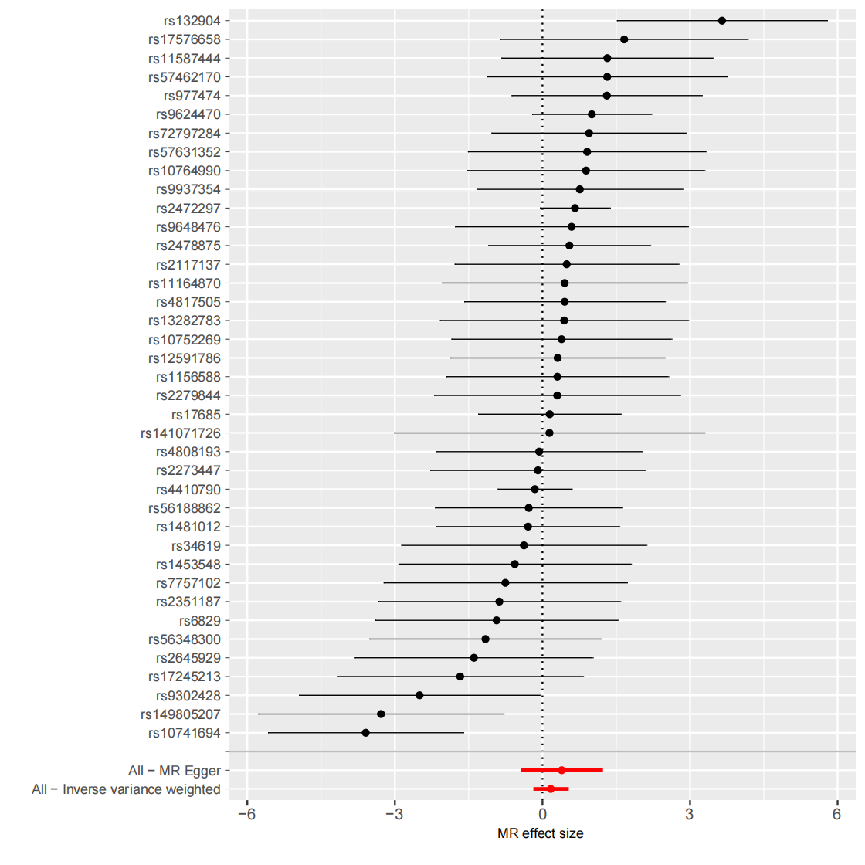
**

**(A)** The scatter plot of the causal effect of tea intake on TB-BMD (age 30-45). Analyses were conducted using the inverse variance weighted and MR-Egger. The slope of each line corresponding to the causal estimates for two methods. **(B)** The funnel plot for the impact of tea consumption on TB-BMD (age 30-45). The background of each SNP was outlined. **(C)** The leave-one-out sensitivity analysis plot for the impact of tea consumption on TB-BMD (age 30-45). The overall error lines remained stable after removing each SNP, reinforcing the reliability of the findings. All: the overall effect without removing SNPs. **(D)** The forest plot for the impact of tea consumption on TB-BMD (age 30-45). Individual SNP effects were computed independently, while the combined impact was assessed through MR-Egger and IVW techniques.

**Supplementary Figure S5.**

**A B**

**
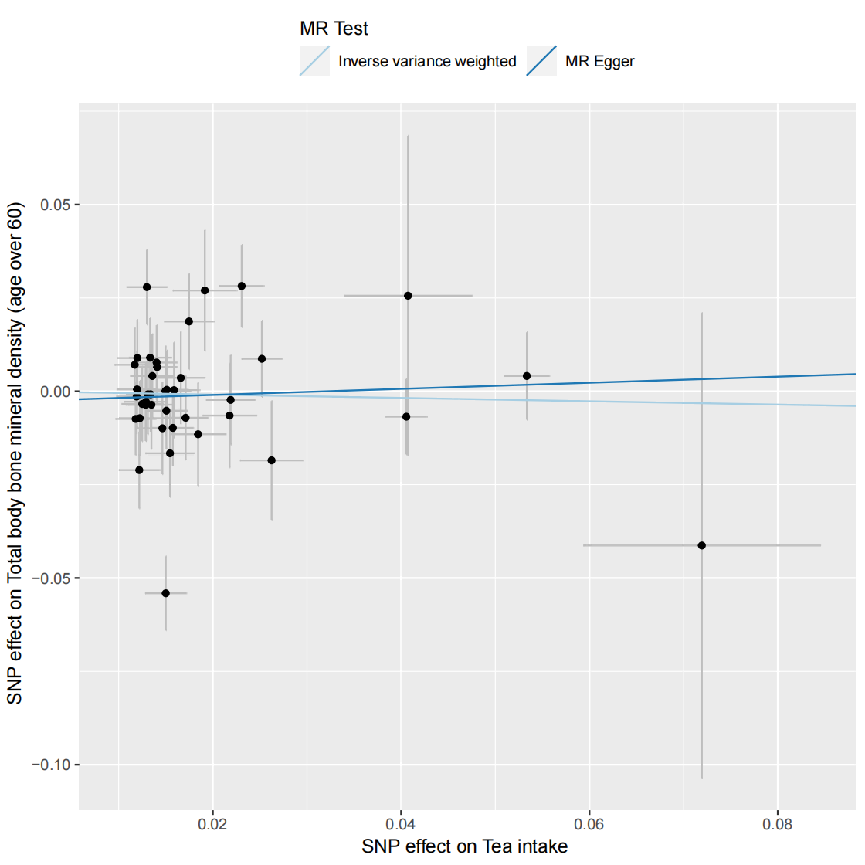

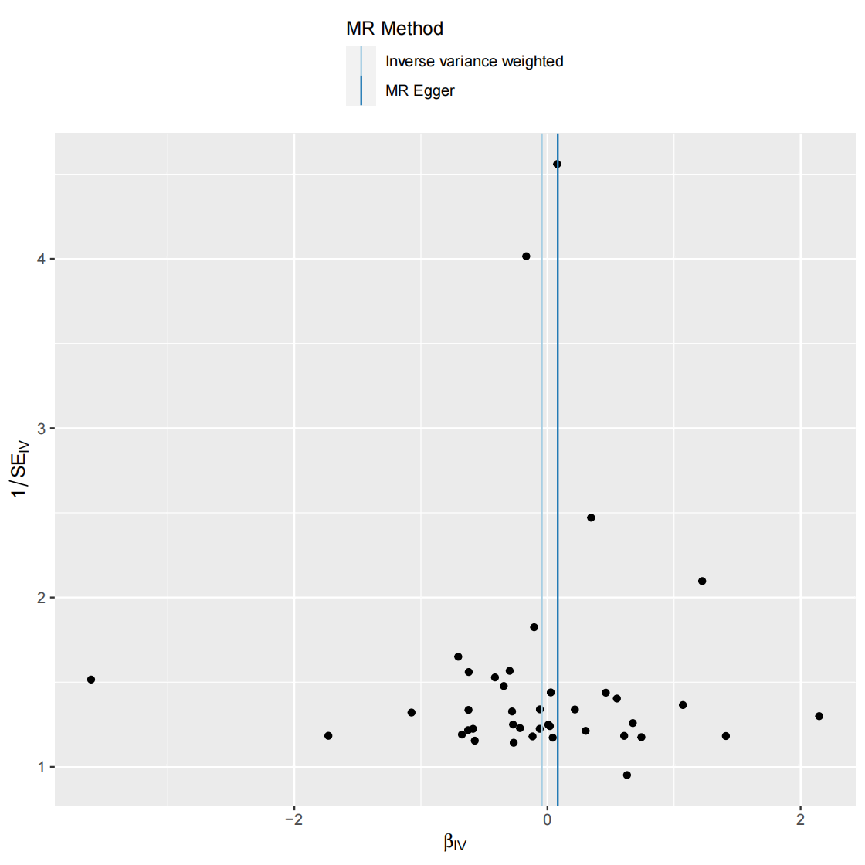
**

**C D**

**
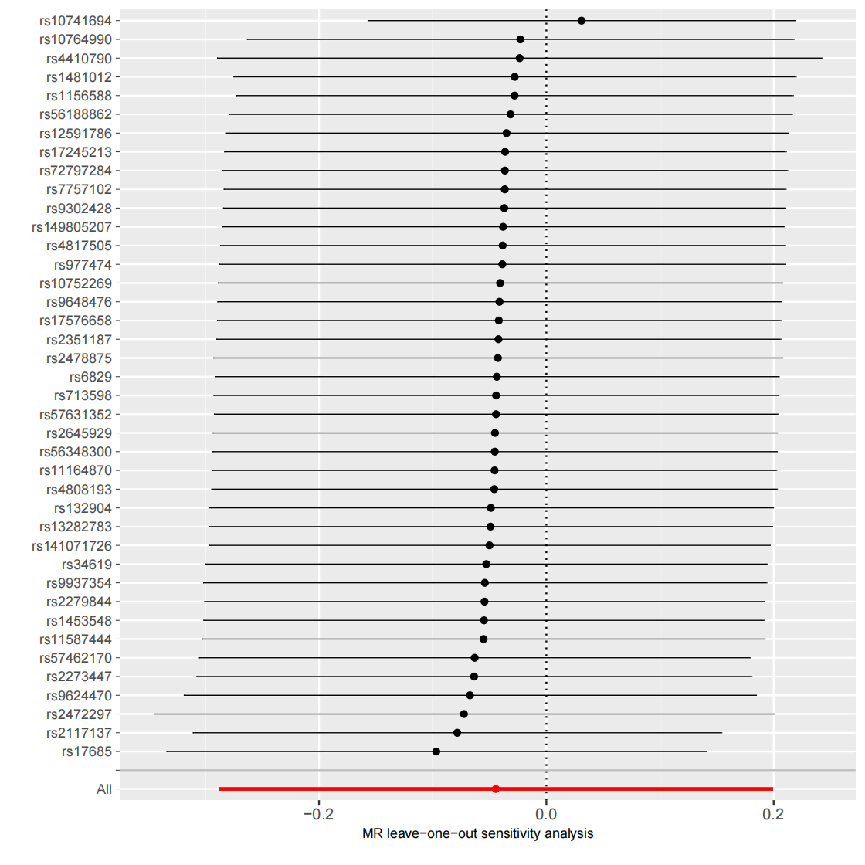

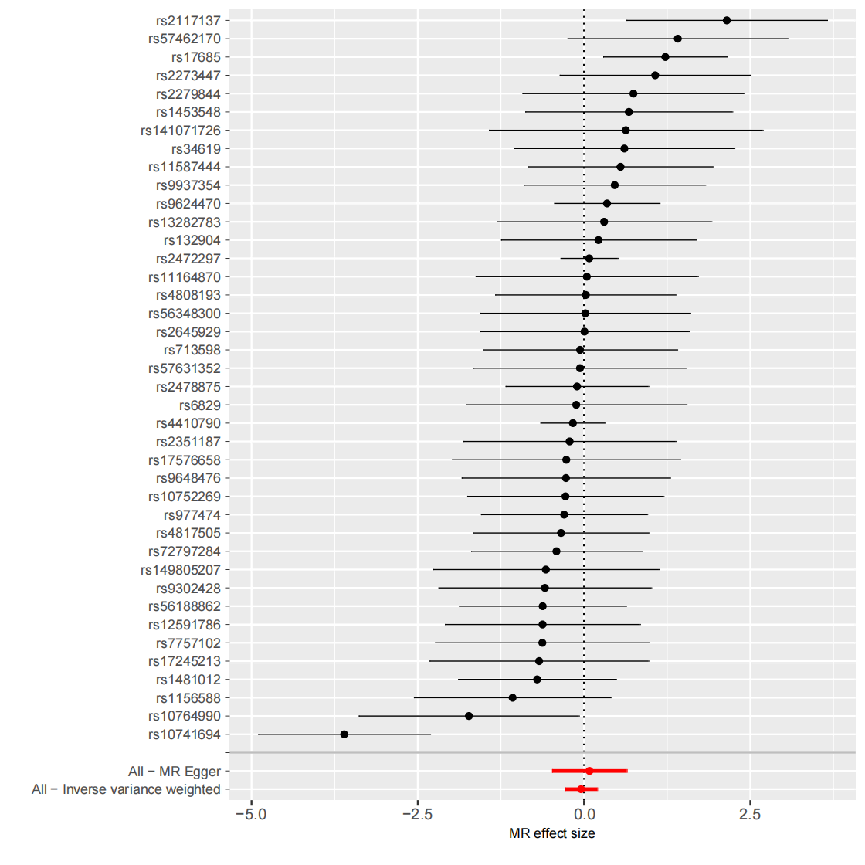
**

**(A)** The scatter plot of the causal effect of tea intake on TB-BMD (age over 60). Analyses were conducted using the inverse variance weighted and MR-Egger. The slope of each line corresponding to the causal estimates for two methods. **(B)** The funnel plot for the impact of tea consumption on TB-BMD (age over 60). The background of each SNP was outlined. **(C)** The leave-one-out sensitivity analysis plot for the impact of tea consumption on TB-BMD (age over 60). The overall error lines remained stable after removing each SNP, reinforcing the reliability of the findings. All: the overall effect without removing SNPs. **(D)** The forest plot for the impact of tea consumption on TB-BMD (age over 60). Individual SNP effects were computed independently, while the combined impact was assessed through MR-Egger and IVW techniques.
